# Structure and dynamics of pteridine reductase 1: the key phenomena relevant to enzyme function and drug design

**DOI:** 10.1007/s00249-023-01677-6

**Published:** 2023-08-22

**Authors:** Joanna Panecka-Hofman, Ina Poehner

**Affiliations:** 1 Division of Biophysics, Institute of Experimental Physics, Faculty of Physics, University of Warsaw, Pasteura 5, 02-093 Warsaw, Poland; 2 School of Pharmacy, University of Eastern Finland, Yliopistonranta 1 C, 70211 Kuopio, Finland

**Keywords:** Pteridine reductase 1, Enzyme regulation, Substrate inhibition, Drug design, Molecular dynamics

## Abstract

Pteridine reductase 1 (PTR1) is a folate and pterin pathway enzyme unique for pathogenic trypanosomatids. As a validated drug target, PTR1 has been the focus of recent research efforts aimed at finding more effective treatments against human parasitic diseases such as leishmaniasis or sleeping sickness. Previous PTR1-centered structural studies highlighted the enzyme characteristics, such as flexible regions around the active site, highly conserved structural waters, and species-specific differences in pocket properties and dynamics, which likely impacts the binding of natural substrates and inhibitors. Furthermore, several aspects of the PTR1 function, such as the substrate inhibition phenomenon and the level of ligand binding cooperativity in the enzyme homotetramer, likely related to the global enzyme dynamics, are poorly known at the molecular level. We postulate that future drug design efforts could greatly benefit from a better understanding of these phenomena through studying both the local and global PTR1 dynamics. This review highlights the key aspects of the PTR1 structure and dynamics relevant to structure-based drug design that could be effectively investigated by modeling approaches. Particular emphasis is given to the perspective of molecular dynamics, what has been accomplished in this area to date, and how modeling could impact the PTR1-targeted drug design in the future.

**Supplementary Information:**

The online version contains supplementary material available at 10.1007/s00249-023-01677-6.

## Introduction

Trypanosomatid parasites are agents causing life-threatening insect vector-borne human diseases, including: African trypanosomiasis (*Trypanosoma brucei*), Chagas’ disease (*Trypanosoma cruzi*), and cutaneous or visceral leishmaniasis (*Leishmania* species) (WHO 2016a, b, c). Currently, trypanosomatid parasites are a major health burden of many developing countries, though leishmaniasis is also a problem in the Mediterranean basin (WHO 2016b). Furthermore, the greenhouse effect can facilitate the appearance of the disease vectors even in currently colder world regions previously not endemic to the disease.

Although there are available therapies for these diseases, including a recent success against sleeping sickness (Dorlo et al. 2012; Nguewa et al. 2005; Priotto et al. 2009; Maxmen 2017), currently used medicines are either affected by issues such as toxicity, parasitic resistance and ineffective drug delivery (Barrett and Croft 2012; Zucca et al. 2013) or can be affected by parasite resistance in the future. Therefore, new anti-trypanosomatid medicines are still needed, and new drug candidates should be searched for.

**Fig. 1:**
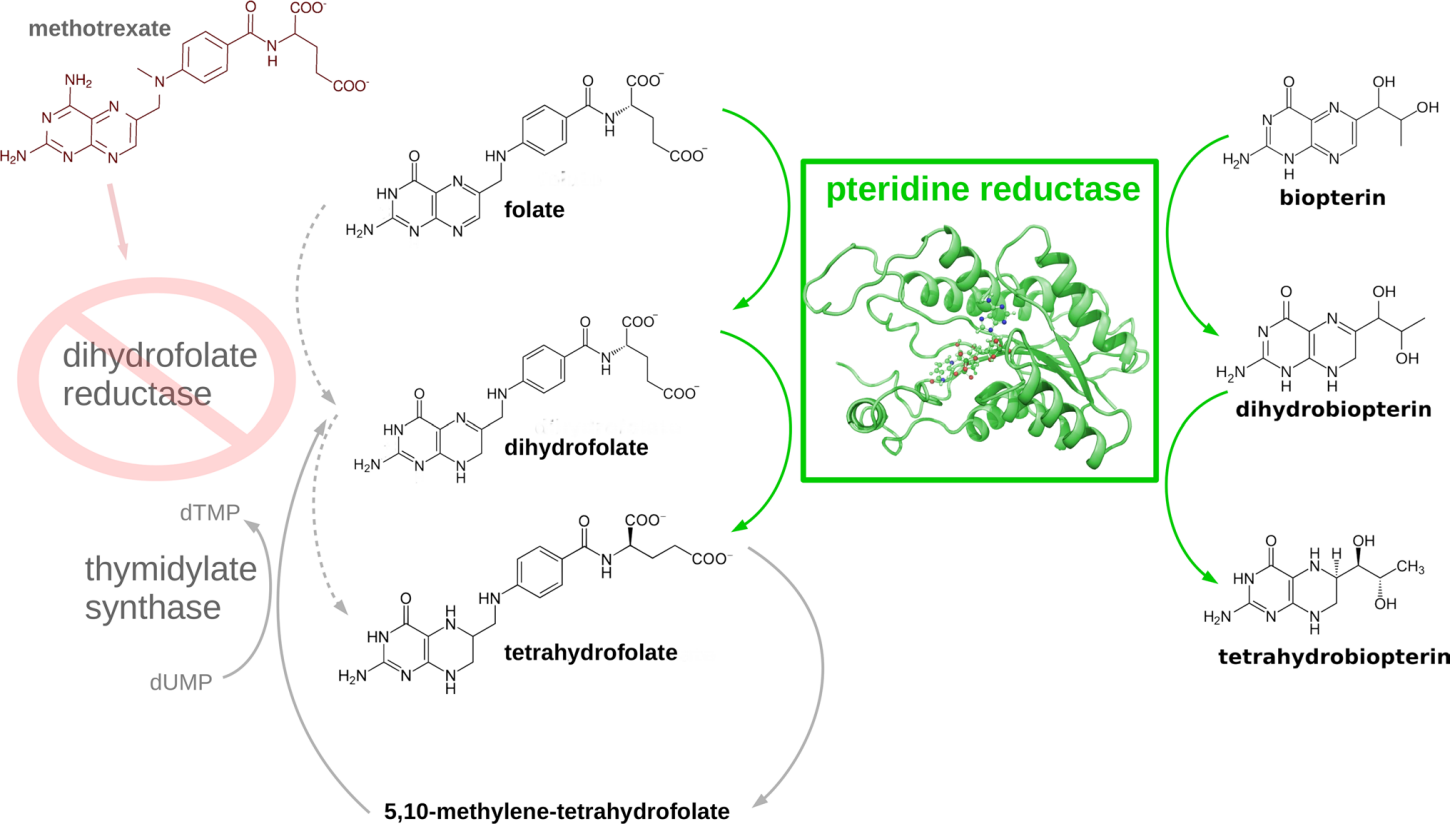
Synopsis of the reviewed portion of the folate pathway with the participating substrates and enzymes, illustrating the role of pteridine reductase 1. An inhibitor of dihydrofolate reductase (DHFR)—methotrexate (MTX)—is shown in the upper left corner. Only a single monomer of the functional PTR1 tetramer is shown as cartoon; the NADP cofactor (NADP$^{+}$) of PTR1 is in ball-and-stick representation. A homology model of the PTR1 enzyme variant from *Trypanosoma cruzi* is displayed (from the work Panecka-Hofman et al. 2017). dUMP is deoxyuridine monophosphate and dTMP—thymidine monophosphate. Figure is adapted from Panecka-Hofman et al. (2017) with permission from the copyright holder

**Fig. 2:**
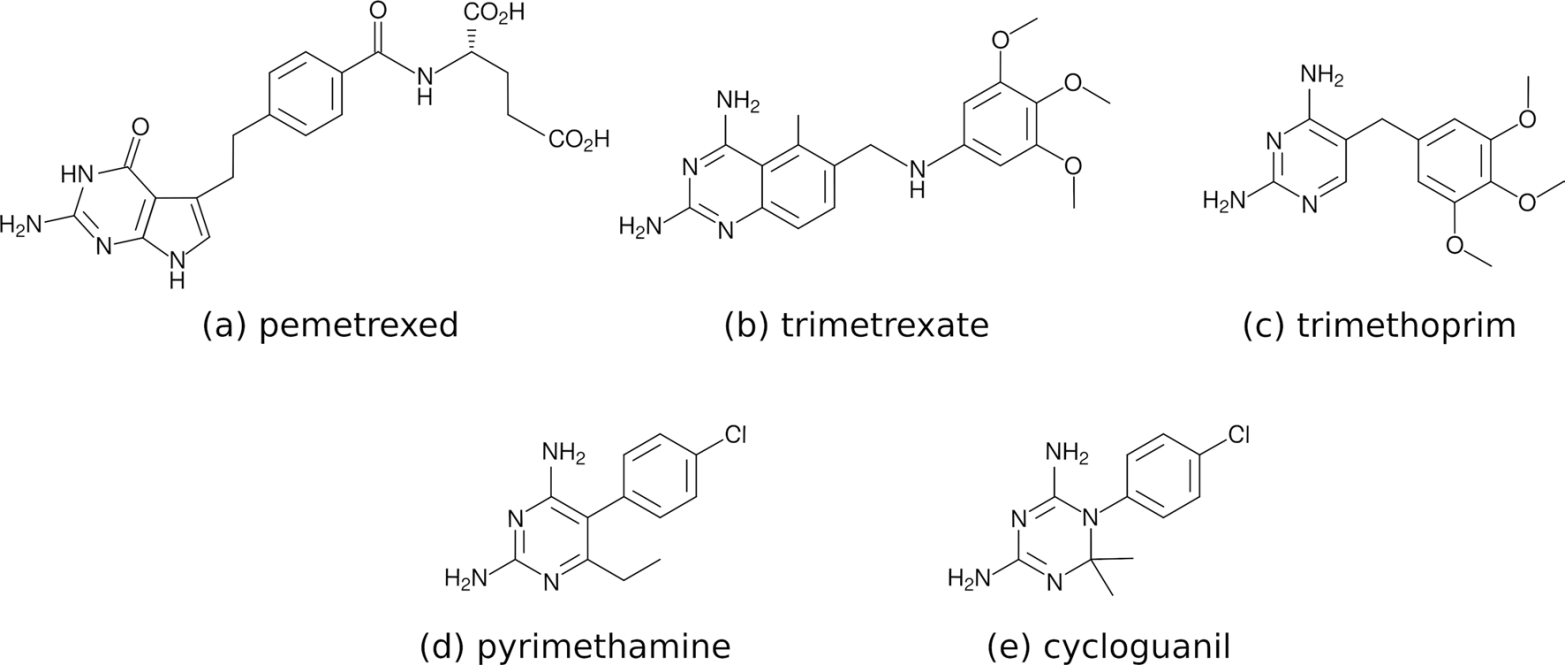
Chemical structures of selected antifolate drugs binding to PTR1 or PTR2

One of the commonly used strategies of compound prioritization is target-based drug design. Unlike mammals, including humans, trypanosomatids are auxotrophs for pterins and folates, which they need to first activate by reduction. One of the explored strategies of anti-trypanosomatid drug design involves targeting the folate and pterin metabolism of these parasites (Fig. 1). The advantage of this approach is the possibility of repurposing existing drugs that target the folate pathway (so-called, antifolates). Examples are drugs targeting a key folate pathway enzyme, dihydrofolate reductase (DHFR), such as the anti-cancer agent methotrexate (MTX, Fig. 1) (Meyer et al. 1950) or anti-parasitic drug—pyrimethamine (Goodwin 1952) (see Fig. 2d). However, trypanosomatids have an additional enzyme—pteridine reductase 1 (PTR1, EC: 1.5.1.33, Fig. 1) that can reduce folates when DHFR is inhibited (Nare et al. 1997; Robello et al. 1997). Therefore, successful anti-trypanosomatid therapy through targeting the folate pathway also requires inhibition of the PTR1 enzyme. PTR1 also performs the reduction of pterins, as shown in Fig. 1. This PTR1-catalyzed reaction occurs with the simultaneous oxidation of the nicotinamide adenine dinucleotide phosphate (NADP) cofactor.^1^

Since the discovery of the PTR1 enzyme in different trypanosomatids, it has been considered an important and promising drug target (Bello et al. 1994; Robello et al. 1997; Dawson et al. 2006). Several PTR1 inhibitors have been designed so far, as reviewed in Calogeropoulou et al. (2019) and Panecka-Hofman et al. (2022). One of the factors that posed a challenge for the drug discovery efforts is incomplete knowledge about the PTR1 dynamics. It is commonly accepted that enzyme dynamics are important for their function and the binding of small molecules, such as substrates and inhibitors (Teilum et al. 2009). In the case of PTR1, in the crystallographic structures, distinct flexibility in the region of the active site can be observed, which likely affects ligand binding. Furthermore, the function of multimers such as the homotetrameric PTR1 may be modulated by ligand binding cooperativity and, in general, by allosteric effects (Wielgus-Kutrowska et al. 2018). The above and other phenomena indicate a potentially important role of dynamics in the PTR1 function. A convenient and effective way of exploring this aspect of biomolecular systems is molecular dynamics (MD) and related techniques. Therefore, in this review, we summarize the knowns and unknowns of the PTR1 dynamics, focusing on the phenomena important for structure-based drug design that could be studied by MD techniques.

## Implications from the PTR1 structural and sequence data for structure-based drug design

### The tertiary and quaternary structure of the PTR1 enzyme

**Fig. 3:**
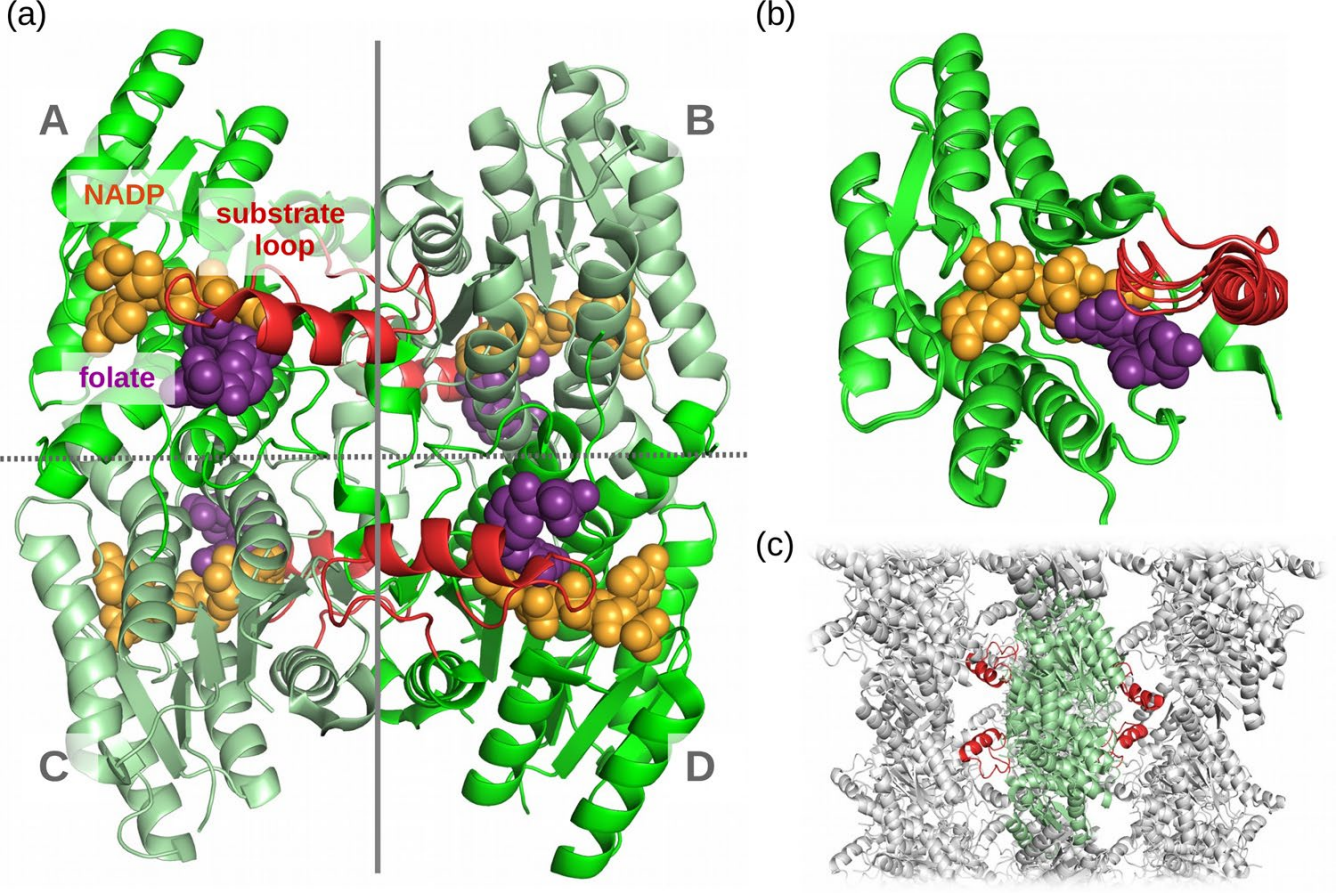
**a** Homotetramer of *T. brucei* PTR1 (PDB code: 3BMC) in the complex with cofactor NADP$^+$ (in orange), and substrate—folate (in purple). The substrate loops are colored red and the two types of differing inter-subunit interfaces are marked by gray lines. The protein is represented as green cartoon; the ligands are in van der Waals representation. Two cofactor and substrate molecules are located on the opposite (hidden) side of the PTR1 homotetramer. **b** Superimposition of the selected *T. brucei* PTR1 crystallographic structures (chain A, PDB codes: 3BMC, 2YHI, 4CMC, 6RX6) showing substrate loop conformational variability; **c** crystal packing interactions of substrate loops in the *T. brucei* PTR1 structure (PDB code: 3BMC). The neighboring homotetramers are in gray

**Fig. 4:**
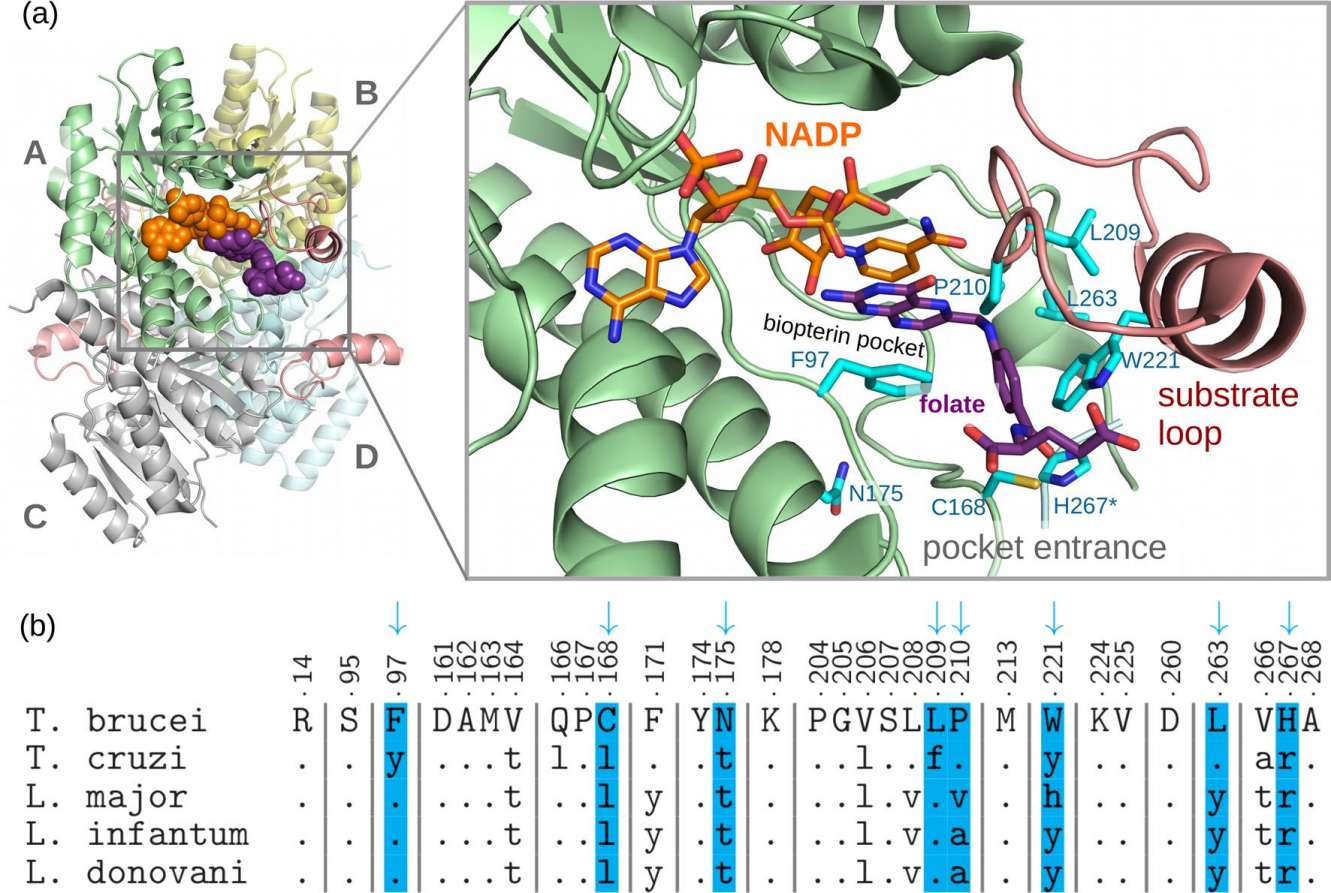
Sequence differences in the binding pocket of different PTR1 variants: **a** binding pocket of *T. brucei* PTR1 (PDB code 3BMC) in the context of the full tetramer (left). NADP (orange) and folate (dark magenta) are shown in chain A, and the substrate loop is colored light red (right). A close-up of the binding pocket, with the most important differing binding site residues shown as cyan sticks; only chain A and a fragment of the C-terminus of chain D are shown (including H267*). **b** Sequence alignment of the binding pocket residues in selected trypanosomatid species, with differing residues displayed in panel (**a**) shaded light blue. The UniProt codes for the aligned sequences: O76290, O44029, Q01782, A4I067, Q6QDB5; sequence numbering for *T. brucei* PTR1 is used. Dots in the alignment imply identity to the reference (top) sequence)

Although PTR1, like DHFR, catalyzes the reduction of folate, it has a completely dissimilar structure and sequence to DHFR. PTR1 belongs to the large family of short-chain dehydrogenases/reductases (Wang et al. 1997). It is a primarily $\alpha$-helical protein (for the *T. brucei* PTR1 structure with PDB code 3BMC (Tulloch et al. 2010): 41% $\alpha$-helical, and 12% $\beta$-sheets) that forms the so-called Rossmann fold characteristic for proteins that bind nucleotide-based cofactors (Hanukoglu 2015). PTR1 forms a homotetramer or, more precisely, a dimer of dimers, with two twofold axes of symmetry, and has two non-equivalent inter-subunit interfaces (see Fig. 3a). The two active sites are located on each side of the homotetramer. It is worth noting that the binding site region in each subunit includes one of the residues from the neighboring subunit (His267 in *T. brucei* or Arg for *L. major* and *T. cruzi*, see Fig. 4). Notably, this may affect the applicability of modeling studies that consider only a single PTR1 monomer. However, the whole PTR1 homotetramer is a relatively large biomolecular system of about 1100 amino acid residues: a single subunit sequence of *L. major* PTR1 contains 288 residues, and *T. brucei* PTR1—268 residues (UniProt accession codes: Q01782 and O76290, respectively). Therefore, modeling of a complete tetramer in solution may be computationally challenging.

### The available crystallographic data and what is missing

Crystallographic structures of PTR1 with substrates and inhibitors are available in the Protein Data Bank (PDB, Sussman et al. (1998)). In particular, as of Jan 21st 2023, there are 76 structures of *T. brucei* PTR1 (18 structures since 2018), 14 structures of *L. major* PTR1 (2 structures since 2018), 2 structures of *T. cruzi* PTR2 (closely related to *T. cruzi* PTR1), and single structures for *L. donovani* and *L. tarentolae* PTR1 (see Tab. S1 in Supplementary Material). A list of selected structures with substrates and classical antifolates is given in Table 1. It should be noted that the above data set could be extended with the help of AlphaFold2 (Jumper et al. 2021) for the other pathogenic trypanosomatid species, which may be helpful in future structure-based inhibitor design.

**Table 1:** Selected crystallographic structures of PTR1 (and related PTR2) from the PDB database with substrates and classical inhibitors (see their 2D structures in Figs. 1 and 2)

Variant	PDB code	Ligand	Resol. (Å)	References
*T. brucei*	3BMC	Folate	2.6	Tulloch et al. (2010)
PTR1	2C7V	Methotrexate	2.2	Dawson et al. (2006)
	2X9V	Trimetrexate	1.3	Dawson et al. (2010)
	2X9G	Pemetrexed	1.1	Dawson et al. (2010)
	6HNC	Cycloguanil	1.5	Landi et al. (2019)
	7OPJ	Pyrimethamine	1.34	Tassone et al. (2021)
*T. cruzi*	1MXH	Dihydrofolate	2.2	Schormann et al. (2005)
PTR2	1MXF	Methotrexate	2.3	Schormann et al. (2005)
*L. major*	1E92	7,8-Dihydrobiopterin	2.2	Gourley et al. (2001)
PTR1	2BF7	7,8-Dihydrobiopterin	2.4	Schüttelkopf et al. (2005)
	2BFP	5,6,7,8-Tetrahydrobiopterin	2.55	Schüttelkopf et al. (2005)
	7PXX	Folate	1.81	Dello Iacono et al. (2022)
	1E7W	Methotrexate	1.75	Gourley et al. (2001)
	2BFM	Trimethoprim	2.60	Schüttelkopf et al. (2005)

Notably, there are relatively few *L. major* PTR1 structures available and they have worse resolution than those of *T. brucei* PTR1 (on average: 2.3 Å vs. 1.8 Å, respectively, considering all the available crystallographic structures as of Jan 21st 2023, Tab. S1 in Supplementary Material). It is interesting to hypothesize about the reason for the overall higher structural fluctuations of *L. major* PTR1 in crystals (vs. *T. brucei* PTR1). In fact, all PTR1 structures contain a few unresolved, poorly resolved or highly fluctuating (in the sense of high B-factors) loop regions (see Fig. S1 in Supplementary Material). The *L. major* PTR1 has one unresolved loop that is absent in the *T. brucei* and *T. cruzi* PTR1 variants (residues 74–80, Fig. S1 in Supplementary Material), protruding from the compact structure of the homotetramer, which may hinder crystallography efforts, and cause overall higher fluctuations of the crystallized *L. major* PTR1. This hypothesis could be verified by building complete, MD-refined, models of the PTR1 variants, and the outcome could support future crystallography efforts for the PTR1–inhibitor complexes.

### The PTR1 substrate loop as a key flexible element

One of the enzyme regions flanking the active site, the so-called substrate loop, is resolved in many chains of the available crystal structures (see Tab. S2 in Supplementary Material), but adopts variable conformations in the crystallographic data (Fig. 3b). It directly contacts most ligands bound in the active site, so it may dynamically affect their binding. Importantly, in many structures, this loop is supported by crystal packing contacts with the PTR1 homotetramer from another crystallographic unit (Fig. 3c). In some cases, when these contacts are absent, fragments of the substrate loops are also unresolved (see Tab. S2 in Supplementary Material). Therefore, it is likely that the level of structural fluctuations of substrate loops observed in crystal structures does not reflect their dynamics in solution.

It has further been proposed that a higher substrate loop flexibility correlates with lower cofactor occupancy (Borsari et al. 2016; Landi et al. 2019; Tassone et al. 2021) while the presence of the cofactor is essential to form the substrate/inhibitor binding site. Furthermore, it has been noted that the interactions between substrate loop, pocket-lining residues and ligands stabilize one another (Borsari et al. 2016; Landi et al. 2019; Tassone et al. 2021). The knowledge of the dynamics of the substrate loop and its impact on the ligand binding site are thus of particular relevance for structure-based drug design.

Modeling the conformational dynamics of PTR1 in solution may thus be helpful in exploring the relevant conformational variability of the substrate loop, and the impact of its dynamics on inhibitor binding.

### Sequence differences between the PTR1 variants and how they affect the binding pocket properties

The PTR1 amino acid sequence is moderately conserved among different trypanosomatids, even in the binding pockets. For instance, the full *T. brucei* PTR1 and *T. cruzi* PTR1 protein sequences are 56% identical, while *T. brucei* PTR1 and *L. major* PTR1—47% (Panecka-Hofman et al. 2017). It is noteworthy that the binding pockets of the PTR1 trypanosomatid variants shown in Fig. 4b have only about 50% identical residues. As evidenced by crystal structures (listed in Table 1), the substrates, folate, biopterin, and their reduced forms, almost do not interact with these differing amino acid residues.

However, most of the currently explored PTR1 inhibitors have differing affinities for different PTR1 variants, e.g., Linciano et al. (2019) and Poehner et al. (2022). The reason lies partly in the aforementioned sequence, and thus—structural, differences of the variant pockets, as summarized in the previous work (Panecka-Hofman et al. 2017). One notable difference is, for example, that Trp221 (*T. brucei*) corresponds to a histidine residue in the *L. major* pocket (Fig. 4). The *T. brucei* PTR1, as a result, features an additional subpocket behind Trp221 (Fig. 4a) that can bind halogenated phenyl moieties (Mpamhanga et al. 2009) (subpocket C in Panecka-Hofman et al. (2017)). Such differences prompted species-specific efforts to support drug discovery efforts against PTR1, such as the evaluation of ligand fragment interaction energies with this subpocket by a quantum-mechanical model (Jedwabny et al. 2017).

A dynamic view of sequence differences and understanding the shape evolution of different PTR1 binding site subpockets over time would enable a more rigorous inclusion of dynamical aspects in the drug discovery process. This could be achieved by analyzing MD-generated pocket conformations with tools such as TRAPP or the TRAPP webserver (Kokh et al. 2013, 2016; Stank et al. 2017).

### Polarity of the PTR1 pockets, solvation and titratable residues

**Fig. 5:**
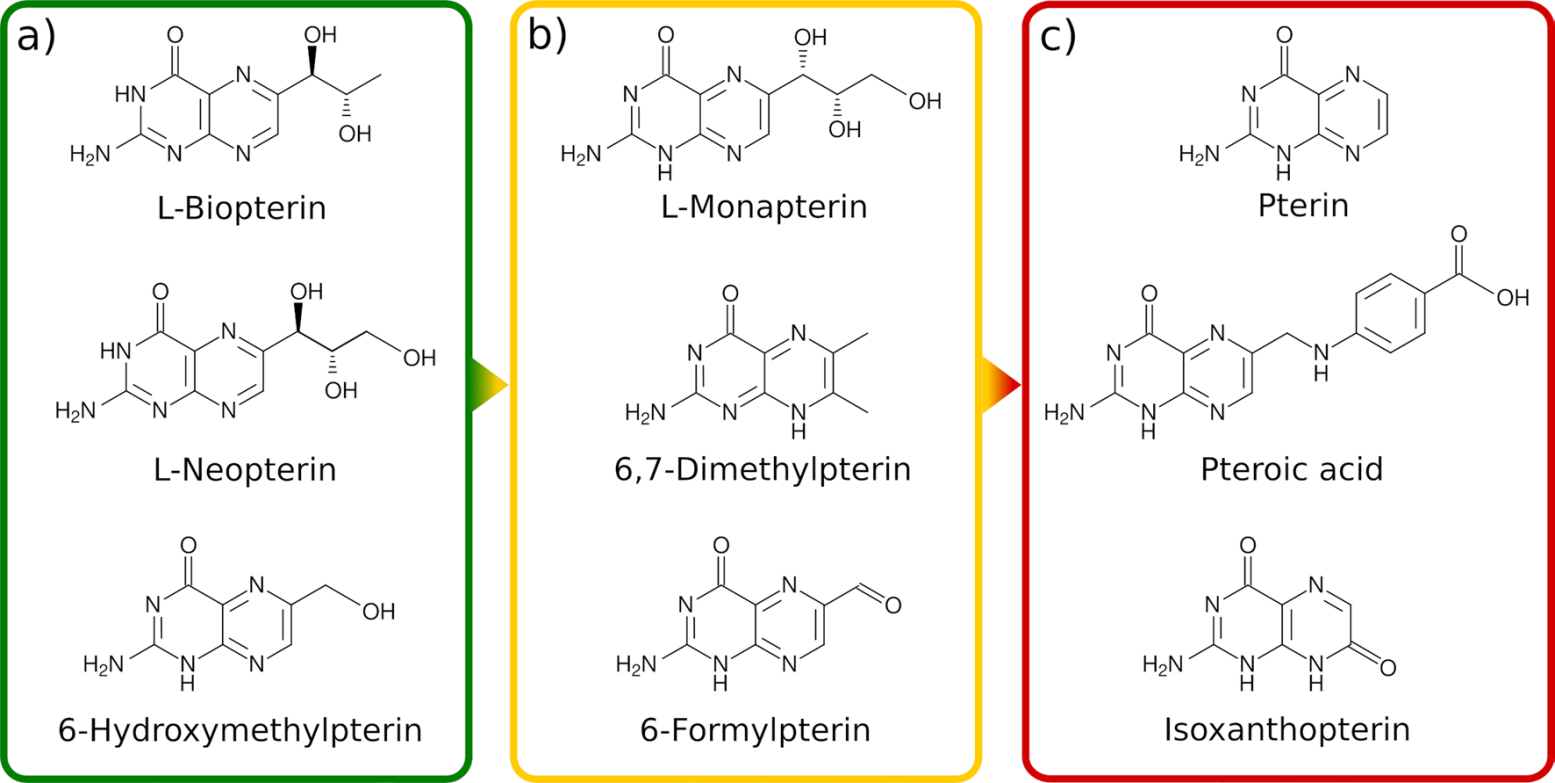
Examples of *L. major* pterin-based nutrients as identified by Nare et al. (1997). The metabolites are divided into **a** ‘good nutrients’ (green frame)—supporting the growth of wild-type *Leishmania* parasites, **b** ‘poor nutrients’ (yellow frame)—non-nutritional for wild-type, but nutritional for ptr1$^-$ mutants transfected with PTR1 plasmids (over-expression of PTR1), and **c** ‘non-nutrients’ (red frame)—non-nutritional under any studied condition

The natural substrates of PTR1, similarly to many antifolates, are relatively polar (Figs. 1, 2 and 5), which also reflects the polar nature of the PTR1 binding pockets (Panecka-Hofman et al. 2017). Consequently, solvation effects and structural waters are important when predicting and analyzing the PTR1–ligand complexes. This aspect has been considered on the basis of crystallographic analysis, e.g., in the design of potent MTX derivatives targeting PTR1 (Poehner et al. 2022). Additionally, bridging water molecules have been previously analyzed in the MD trajectories of specific *L. major* PTR1–inhibitor complexes (Istanbullu et al. 2020). It is not however clear how the hydration properties of the PTR1 pocket depend on the bound ligand and on the PTR1 variant. Crystallographic data, for example, suggest that the specific conserved water in *T. brucei* PTR1 may be replaced by submicromolar benzimidazole derivatives displaying non-classical binding modes (Mpamhanga et al. 2009) (Figure S2 in Supplementary Material). When attempting to routinely exploit solvation effects for anti-PTR1 drug discovery, understanding the nature of such conserved structural waters and the energetic implications of their replacement would be indispensable knowledge (Spyrakis et al. 2017). To further extend on those observations and exploit solvation effects routinely in anti-PTR1 drug discovery, MD simulations of PTR1 could help in elucidating ensemble-averaged solvation patterns and water residence times, and discern ‘hot’ from ‘cold’ waters in the binding pockets (Spyrakis et al. 2017).

Furthermore, His267 and other histidines in the PTR1 active sites, such as His241 in the *L. major* variant (corresponding to Trp221 in *T. brucei*), have unclear protonation state. Notably, the optimal pH for both biopterin and folate reduction is acidic (4.7 and 6.0, respectively) (Nare et al. 1997), which may also additionally bring up the question about the protonation state of the active site Asp (161 in *T. brucei* and 181 in *L. major*) that was suggested to be a catalytic residue (Gourley et al. 2001).

The effect of different pH values on the PTR1 activity and consequences for ligand binding could be investigated to ensure the use of a biologically relevant receptor protonation state in drug design. The systems with specific protonation states could be studied with classical MD or the balance between ligand and protein protonated and deprotonated states could be investigated with constant-pH MD approach (Martins de Oliveira et al. 2022).

## The phenomena related to the PTR1 function that require dynamical insights, and their relevance for drug design

### The PTR1 substrate selectivity

Apart from folate and biopterin, PTR1 can catalyze the reduction of other pterin substrates with differing efficiencies (Nare et al. 1997) (see Fig. 5). Notably, differences in the catalytic activities of PTR1 towards oxidized and reduced forms of folate and pterin substrates have been reported (Bello et al. 1994; Robello et al. 1997). For instance, early reports state that *L. major* PTR1 shows the highest catalytic activities with fully oxidized biopterin, intermediate for oxidized folate, and the lowest for dihydrobiopterin and dihydrofolate (Bello et al. 1994). Moreover, for *L. major* PTR1, Nare et al. (1997) reported differing activities for different pterin forms. For instance, it has been shown that *L. major* PTR1 is poorly active for the so-called quinoid form of dihydrobiopterin (Nare et al. 1997).

The molecular origin of this substrate selectivity of PTR1 has not been explored yet, but it emphasizes how subtle differences in the interaction pattern translate into notable activity differences. Studies of the above phenomena, e.g., with classical MD, will not only improve our understanding of the PTR1 enzyme activity but also provide valuable knowledge for anti-PTR1 drug design since PTR1 inhibitors known to date are commonly competitive inhibitors and many inhibitor structures were inspired by the chemical scaffold of known substrates (e.g., compare MTX and folate in Fig. 1).

### The substrate inhibition phenomenon

Interestingly, it has been observed for *L. major* PTR1 and *T. cruzi* PTR1 that, at certain concentration, PTR1 enzymes are inhibited by the semi-product of the catalyzed reaction (dihydrobiopterin and dihydrofolate) (Nare et al. 1997). As noted by Gourley et al. (2001), it is striking that substrate inhibition is caused only by dihydrobiopterin and dihydrofolate, and not by their oxidized equivalents. Therefore, this effect at least partly depends on the specific interactions of the ligand in the binding site. However, more long-range factors, like electrostatics and overall PTR1 homotetramer dynamics, may also play a role. It is possible that the PTR1 binding sites are dynamically coupled, thus exerting the substrate inhibition effect at sufficient concentration, when all sites are occupied. However, the range and importance of dynamical cooperativity between the PTR1 subunits are unknown. Investigation of the PTR1 homotetramer dynamics at different spatial and temporal scales could help in explaining these phenomena. However, exploring the global dynamics of the PTR1 system would require classical MD approaches significantly exceeding microsecond time scales or effective enhanced sampling approaches.

### The unexplored case of the *T. cruzi* PTR1 and PTR2

In contrast to *L. major* and *T. brucei*, the folate pathway of *T. cruzi* is relatively unexplored. Notably, PTR1 was found only in the insect stage (and not in the human stage) of the *T. cruzi* parasite (Robello et al. 1997). Furthermore, there exist no crystallographic structures of *T. cruzi* PTR1, only structural data for the related protein, *T. cruzi* PTR2, are available (Schormann et al. 2005). The *T. cruzi* PTR2 enzyme is catalytically active towards dihydrofolate and dihydrobiopterin, and inactive for their fully oxidized forms (Senkovich et al. 2003), which is in contrast to *T. cruzi* PTR1 that is more active towards the oxidized forms (Robello et al. 1997). The catalytic activity difference of *T. cruzi* PTR1 vs. *T. cruzi* PTR2 is likely related to the structural differences, though the *T. cruzi* PTR1/*T. cruzi* PTR2 sequence difference is not striking: only one amino acid differs in the active site (Phe vs. Leu, respectively, at position 209, see Fig. 4) and there are 9 residue differences in total (Senkovich et al. 2003). Furthermore, according to Senkovich et al., PTR2 is less sensitive to MTX than PTR1, with $IC_{50}$ of 5.5 $\upmu$M for *T. cruzi* PTR2 vs. 0.112 $\upmu$M for *T. cruzi* PTR1 (Schormann et al. 2005).

Therefore, classical MD could help in exploring what is the reason for the *T. cruzi* PTR1/*T. cruzi* PTR2 activity and inhibitor binding differences, and if it can be explained by the protein dynamics.

### Is cofactor and ligand binding to PTR1 cooperative?

The PTR1-catalyzed reaction occurs with the simultaneous oxidation of the NADPH cofactor (Fig. 1). It is however unclear how the NADP$^+$ reduction occurs and, especially if NADP$^+$ needs to leave its binding site in the PTR1 homotetramer, as suggested in some papers (Luba et al. 1998; Gourley et al. 2001). There exists only one crystallographic structure of apo-PTR1 (*L. donovani*, PDB code 1P33), that contains no cofactor (Barrack et al. 2011). The active sites of the enzyme are disordered and substrate loops are partially missing, but it is unknown whether this is the reason or the cause of the cofactor absence. Notably, for FabG, one enzyme with certain similarity to PTR1 (over 30% sequence identity and sharing the same homotetrameric quaternary structure with the NADP-binding Rossmann fold), the structures lacking the cofactor remain stable (e.g., PDB code 4AFN), and in some FabG structures one site lacks one cofactor (e.g., *P. aeruginosa* FabG, PDB code 4AG3, Fig. S3a in Supplementary Material), which is not observed for PTR1. Furthermore, there are some reports of negative cooperativity of NADP binding to the tetrameric *E. coli* FabG enzyme (Price et al. 2004), which prompts the question whether ligand binding to PTR1 may also be cooperative or anti-cooperative. It is noteworthy that in some crystal structures of PTR1, only three of the four sites in the homotetramer are occupied by inhibitors, while one site remains empty, which could be consistent with the hypothesis of negative cooperativity, as briefly discussed in Panecka-Hofman et al. (2022). Since the cooperativity phenomenon is related to the global enzyme dynamics, further insights could be provided by extensive sampling of the PTR1 homotetramer conformations, similarly as in the case of substrate inhibition.

## Dynamical aspects of the PTR1-targeted drug design

### Flexibility of the PTR1–inhibitor complexes

So far, there have been several structure-based attempts to design PTR1-specific inhibitors. These include older works (e.g., Mpamhanga et al. (2009); Spinks et al. (2011); Cavazzuti et al. (2008); Corona et al. (2012)) and the follow-up work in the more recent EU-funded project NMTrypI (New Medicines for Trypanosomatidic Infections, https://fp7-nmtrypi.eu/), in which many folate pathway and PTR1 inhibitors were designed and tested against enzymes and parasites by combined computational and experimental approaches (Moraes et al. 2019). Many of the designed PTR1 inhibitors are inspired by the structures of the known drugs, antifolates, including the aforementioned MTX (see Fig. 1) (Cavazzuti et al. 2008; Corona et al. 2012; Poehner et al. 2022). However, many antifolates (Fig. 2), similarly to folate, have flexible ‘tails’, i.e., moieties with many rotational degrees of freedom. The high B-factors or missing fragments of these in the crystal structures of PTR1 complexes suggest their significant flexibility and the role of entropy in binding (Poehner et al. 2022; Pöhner 2020). Notably, these tails interact with the likely flexible substrate loops (Fig. 3b). Therefore, a detailed investigation of the dynamics of antifolate-PTR1 complexes would be helpful in elucidating enzyme–inhibitor dynamical interaction patterns, and thus could help in future PTR1 inhibitor optimization.

### Modeling of the PTR1–inhibitor complexes dynamics with non-MD approaches

The initial works to design PTR1 inhibitors, including the outcomes of the NMTrypI project, have shown that there is a need to investigate the PTR1 dynamics to improve the understanding of the experimental data, in particular, of the structure-activity relationships of PTR1-targeting compounds.

One of the less time-consuming methods to include receptor flexibility is induced-fit docking. For example, such an approach allowed for explaining the distinct activity profile of flavonoid-based inhibitors in different variants of PTR1 (Borsari et al. 2016). This highlights the importance of pocket flexibility for the PTR1-targeted drug design. Useful insights into the PTR1 dynamics were also provided as part of a first comprehensive comparative study of the folate pathway enzyme crystal structures in trypanosomatids and humans (Panecka-Hofman et al. 2017). The analysis with the TRAPP tool (Kokh et al. 2013; Stank et al. 2017) suggested that conformational adaptation may play a significant role in the PTR1 ligand binding. Furthermore, normal modes analysis of *T. brucei* PTR1 has shown concerted movements of the four substrate loops in the homotetramer (Wodak et al. 2019).

### Modeling of the PTR1 dynamics with MD techniques: the current status and the road ahead

There already exist several studies that have applied MD techniques to the PTR1 complexes with inhibitors (Kimuda et al. 2019; Istanbullu et al. 2020; Leite et al. 2016, 2017; Cardona et al. 2021; Herrera-Acevedo et al. 2021; Kapil et al. 2019; Bekhit et al. 2022; Mohamed et al. 2023; Ibrahim et al. 2023; Boakye et al. 2023) (some of these have been recently reviewed in Panecka-Hofman et al. (2022)). The time scales of MD simulations were in the range 20–200 ns, and they were mostly performed for PTR1 monomers. The primary aim of these studies was to validate the stability of the inhibitor binding modes in structure-based drug design approaches. Typically, the binding modes from virtual screening workflows were indeed found to be stable in the reported simulations. Some of the studies have also used MMGBSA or MMPBSA techniques to estimate inhibitor binding free energies (Kimuda et al. 2019; Istanbullu et al. 2020; Herrera-Acevedo et al. 2021; Boakye et al. 2023).

Despite the focused applicability domain and limited time scale, some of the studies provided interesting observations regarding the PTR1 dynamics, such as the notion of substrate loop flexibility (Kimuda et al. 2019). They also have shown that all the ligand-bound *T. brucei* PTR1 systems were more compact when compared to the ligand-free systems, and, in general, all systems were becoming more compact in the course of the simulations. It is not however clear if the homotetrameric PTR1 systems would behave similarly to the simulated monomers due to the constraints imposed by the interactions with the other subunits. On the more local scale, Istanbullu et al. (2020) additionally mentioned the importance of solvation patterns for stabilization of thiazolopyrimidine derivatives in the *L. major* PTR1 active site.

In most of the reported MD studies, only the PTR1 monomers were subjected to MD simulations. Such an approach may be sufficient for studying the stability of the inhibitor-PTR1 complexes, though one has to note that the missing C-terminus from the other subunit in the PTR1 homotetramer (containing basic residues His or Arg, see Fig. 4) may affect the results. Furthermore, such relatively short-time-scale studies obviously do not allow for investigating the global dynamics of the functional PTR1 homotetramer. With such an objective, much longer simulations would be needed, and considering the limitations of computational resources—enhanced sampling approaches. Implicit-solvent non-equilibrium simulations such as the Rotamerically Induced Perturbation (RIP) method (Ho and Agard 2009) have already been used to investigate long-range dynamics of PTR1, and indicated coupling of loops in the *L. major* PTR1 homotetramer (Wodak et al. 2019). Also, the modified method variant, Langevin-RIP (Kokh et al. 2016) can be considered to relatively quickly explore the flexibility of substrate loop on the micro- to millisecond time scale. The related simulation technique, Anisotropic Thermal Diffusion (Ota and Agard 2005) could be also used to analyze the kinetic energy flows between residues in the PTR1 homotetramer, and thus help in deciphering the dynamic inter-residue networks potentially involved in regulating the PTR1-related global-dynamics phenomena.

Alternatively, the Gaussian accelerated MD (Wang et al. 2021) could be used to sample the conformations of the PTR1 homotetramers. This method has been used for exploring global conformational transitions of such large systems as CRISPR-Cas9 (over 1300 residues, the approach combined with targeted MD) (Palermo et al. 2017) or for detecting cryptic pockets in the SARS-CoV-2 main protease (over 600 residues for a dimer) (Sztain et al. 2020).

In summary, detailed and more general knowledge about the PTR1 dynamical properties on the local and global scale in the context of its function and structure-based drug design is still needed.

### The prospect of allosteric inhibitor design

The known inhibitors of PTR1 are typically competitive, targeting the substrate binding site. However, one might also consider designing allosteric inhibitors, which could help avoid the off-target issues inherent to the inhibitor design based, e.g., on classical antifolates (Panecka-Hofman et al. 2022). Notably, for the aforementioned FabG enzyme (from *P. aeruginosa*), sharing structural characteristics with PTR1, a series of allosteric inhibitors have been discovered (Cukier et al. 2013). The binding mode and mechanism of action have been found with the help of crystallography (Fig. S3 in Supplementary Material): the compounds bound to the inter-subunit interface (marked by gray dotted line in Fig. 3a for PTR1), and inactivated the FabG enzyme by disabling NADP binding due to the significant perturbation of the entire homotetramer structure. The interesting question would be if a similar conformational transition could also occur for PTR1 with the appropriately redesigned inhibitors. This could be more easily answered with the knowledge of long-range and long-time-scale PTR1 dynamics, e.g. with the simulation methods proposed above, and how those compare to FabG.

## Summary and outlook

In this review, we highlighted the current status of structural and dynamical studies of PTR1 and proposed what aspects of the PTR1 function and properties could be modeled in the future to support structure-based drug design against the enzyme.

The significant amount of crystallographic data for PTR1 provided structural knowledge about the enzyme and served as a basis for many inhibitor design studies. So far, the PTR1-targeted structure-based drug design has been focused mostly on (but not limited to) ‘static’ approaches such as 3D-QSAR, virtual screening, or molecular docking simulations, reviewed in Calogeropoulou et al. (2019) and Panecka-Hofman et al. (2022). This resulted in the design of potent inhibitor series with up to picomolar activities [for pteridine derivatives targeting *T. brucei* PTR1 (Poehner et al. 2022)]. Despite many successes, the research on PTR1 inhibitors so far has shown general problems, such as off-target selectivity and compound transport within the parasite cell and host organism (Panecka-Hofman et al. 2017; Poehner et al. 2022). At the molecular level, the difficulties in structure-based design resulted from an insufficient understanding of the PTR1 structural and dynamical properties and their connection to the PTR1 function.

Although several MD studies have already been performed as part of drug design workflows to test the stability of the selected PTR1 inhibitor binding modes (reviewed here and in Calogeropoulou et al. (2019) and Panecka-Hofman et al. (2022)), to the best of our knowledge, the dynamical properties of PTR1 have not yet been extensively studied in a more general way in the context of the enzyme function and inhibitor design. Such research would offer novel insights into the dynamics-dependent properties of the PTR1 enzyme and its complexes, the importance of which can already be seen from the crystallographic data and the structure-based drug design attempts performed so far. Dynamical studies at different time scales could provide a better understanding of the PTR1 activity and its interactions with inhibitors, which are often flexible, similarly to one key substrate—folate. The knowledge about dynamical differences between PTR1 variants would likely support multi-variant PTR1 inhibitor design, since the ‘static’ approaches in some cases fail to explain inhibitor activity differences between different PTR1 variants (Borsari et al. 2016), which is important for targeting multiple trypanosomatid species.

Knowledge about the PTR1 dynamics would be also methodologically helpful for computational drug design efforts. In particular, more detailed insights are needed into the PTR1 binding pocket solvation and protonation states, since PTR1 has a relatively polar and solvent-exposed binding pocket (Panecka-Hofman et al. 2022). These have been identified as problematic issues in the PTR1-centered ligand–protein docking studies (Poehner et al. 2022; Pöhner 2020). Second, the knowledge about the PTR1 binding pocket flexibility may support future ligand docking simulations, since PTR1 pockets are relatively open (Panecka-Hofman et al. 2022), and thus some of the residues possess significant conformational freedom. They could inform which residues should be treated as flexible in induced-fit docking approaches and provide starting conformations for ensemble docking simulations. Finally, the study of the PTR1 dynamics may also suggest alternative drug design strategies, involving, for instance, the targeting of protein inter-subunit interfaces, as done for a protein similar to PTR1–FabG (Cukier et al. 2013).

In summary, the described PTR1-related phenomena could effectively be studied by computational methods. The results could significantly boost the approaches to PTR1-specific structure-based drug design and help to verify important hypotheses about the PTR1 function. Whether by resulting in novel PTR1 inhibitors or by serving as a role model for the adaptation of a more holistic and dynamic view on parasite enzymes as drug targets, this would, ultimately, be of help to the anti-trypanosomatid drug development and the fight against the devastating human parasitic diseases caused by trypanosomatids.

### Supplementary Information

Below is the link to the electronic supplementary material.

Supplementary file 1 (pdf 22304 KB)

## Footnotes

1 Further referred to as either NADP$^+$ or NADPH when its specific oxidation state is discussed.
