# Supplementary material for: Structure and dynamics of pteridine reductase 1: the key phenomena relevant to enzyme function and drug design
Source: Eur Biophys J. 2023 Aug 22;52(6-7):521–32. doi: 10.1007/s00249-023-01677-6 (PMC10618315; doi:10.1007/s00249-023-01677-6)
Supplement: Supplementary file 1 — (pdf 22304 KB) [file 249_2023_1677_MOESM1_ESM.pdf]

Electronic Supplementary Material: Structure and dynamics of pteridine reductase 1: the key phenomena relevant to enzyme function and drug design

Joanna Panecka-Hofman[1*] and Ina Poehner[2]

[1*]Division of Biophysics, Institute of Experimental Physics, Faculty of Physics, University of Warsaw, Pasteura 5, Warsaw, 02-093, Poland.
[2]School of Pharmacy, University of Eastern Finland, Yliopistonranta 1 C, Kuopio, 70211, Finland.

*Corresponding author(s). E-mail(s): joanna.panecka@uw.edu.pl;

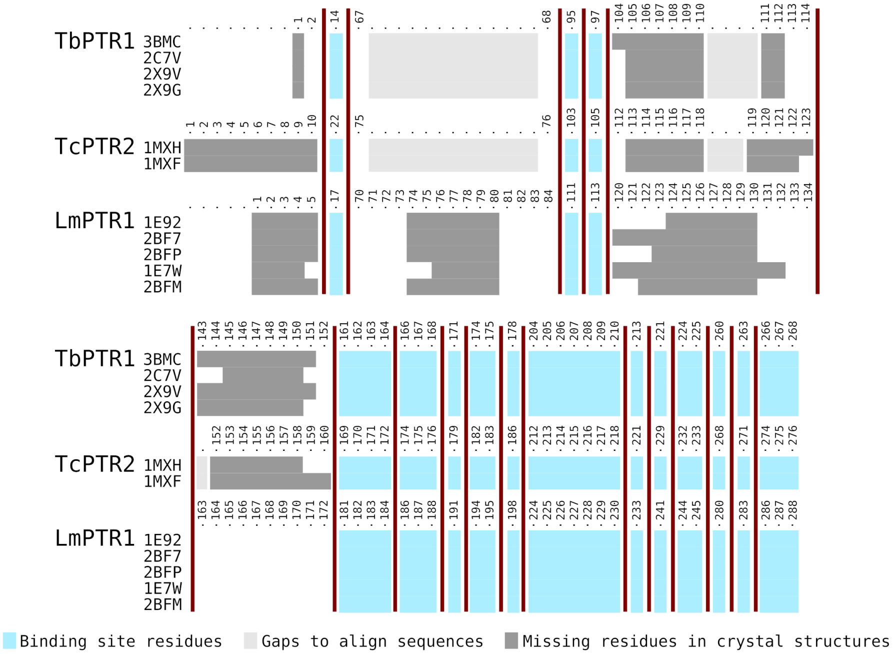

Fig. S1: Overview of missing residues (dark gray) and binding site residues (light cyan, defined as in Fig. 4 in the main text) for the discussed structures of *T. brucei* PTR1 (TbPTR1), *T. cruzi* PTR2 (TcPTR2) and *L. major* PTR1 (LmPTR1) in the aligned protein sequences. Light gray fields indicate gaps introduced in the multiple sequence alignment. Residue numbers for each protein are given above the corresponding bar chart.

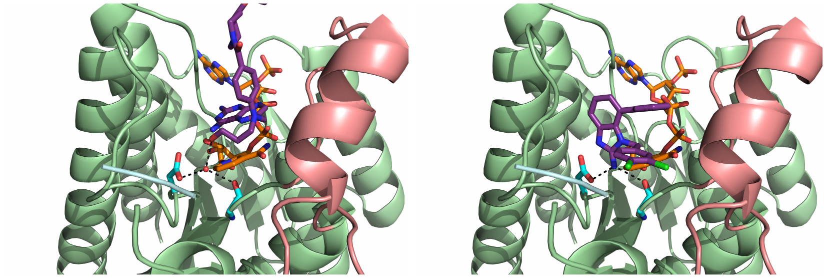

(a) (b)

Fig. S2: (a) A crystallographic water, bridging between Asp161 and Gly205, present in most crystallographic complexes of *T. brucei* PTR1 (with a pteridine derivative, PDB code: 6RX6 Poehner et al (2022)) is displaced by (b) the compound showing a non-classical binding mode (1-(3,4-dichlorobenzyl)-7-phenyl-1h-benzimidazol-2-amine, PDB code: 2WD8) Mpamhanga et al (2009). Color-coding is based on Figure 4 in the main text with inhibitors colored in purple.

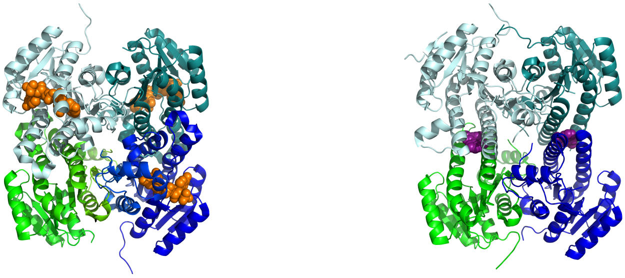

(a) (b)

Fig. S3: *P. aeruginosa* FabG structures: a) with NADPH bound (in 3 sites, in orange; PDB code: 4AG3), and b) with allosteric inhibitor bound (in purple, PDB code: 4BO4). The orientation of the FabG structures is similar to that of *T. brucei* PTR1 in Fig. 3 in the main text.

Table S1: The PDB codes of *T. brucei* and *L. major* PTR1 structures as of Jan 21st 2023.

**Variant**	**PDB codes**
T. brucei	2C7V, 2VZ0, 2WD7, 2WD8, 2X9G, 2X9N, 2X9V, 2YHI, 2YHU, 3BMC, 3BMN, 3BMO, 3BMQ, 3GN1, 3GN2, 3JQ6, 3JQ7, 3JQ8, 3JQ9, 3JQA, 3JQB, 3JQC, 3JQD, 3JQE, 3JQF, 3JQG, 3MCV, 4CL8, 4CLD, 4CLE, 4CLH, 4CLO, 4CLR, 4CLX, 4CM1, 4CM3, 4CM4, 4CM5, 4CM6, 4CM7, 4CM8, 4CM9, 4CMA, 4CMB, 4CMC, 4CME, 4CMG, 4CMI, 4CMJ, 4CMK, 4WCD, 4WCF, 5IZC, 5JCJ, 5JCX, 5JDC, 5JDI, 5K6A, 6GCK, 6GCL, 6GCP, 6GCQ, 6GD0, 6GD4, 6GDO, 6GDP, 6GEX, 6GEY, 6HNC, 6HNR, 6HOW, 6RX0, 6RX5, 6RX6, 6TBX, 7OPJ
L. major	1E7W, 1E92, 1W0C, 2BF7, 2BFA, 2BFM, 2BFO, 2BFP, 2QHX, 3H4V, 5L42, 5L4N, 6RXC, 7PXX

Electronic Supplementary Material

Table S2: *T. brucei* and *L. major* PTR1 PDB structures as of Jan 21st 2023 that have residues missing in one or more substrate loops. The names of chains with missing residues are also provided.

PDB code	chain
T. brucei	
3jq6	B
3jq7	C
3jq8	A, C
4wcd	C
4wcf	B, C
5izc	C
5jcj	C
5jcx	C
5jdc	B, C
5jdi	C
5k6a	C
6gd0	C
6how	C
6rx0	C
6rx5	C
6rx6	C
6tbx	C
7opj	C
L. major	
1e7w	B
1e92	C, D
1w0c	C, D
2bf7	C, D
2bfa	C, D
2bfm	C, D
2bfo	C, D
2bfp	C, D
2qhx	C, D
3h4v	C, D
5l42	C, D
5l4n	A, B
6rxc	A, B, C, D
7pxx	C, D

References

Mpamhanga CP, Spinks D, Tulloch LB, et al (2009) One scaffold, three binding modes: novel and selective pteridine reductase 1 inhibitors derived from fragment hits discovered by virtual screening. J Med Chem 52(14):4454–4465. https://doi.org/10.1021/jm900414x, URL http://dx.doi.org/10.1021/jm900414x

Poehner I, Quotadamo A, Panecka-Hofman J, et al (2022) Multitarget, selective compound design yields potent inhibitors of a kinetoplastid pteridine reductase 1. J Med Chem 65:9011–9033. https://doi.org/10.1021/acs.jmedchem.2c00232
